# The novel *MAPT* mutation K298E: mechanisms of mutant tau toxicity, brain pathology and tau expression in induced fibroblast-derived neurons

**DOI:** 10.1007/s00401-013-1219-1

**Published:** 2013-11-30

**Authors:** Mariangela Iovino, Ulrich Pfisterer, Janice L. Holton, Tammaryn Lashley, Robert J. Swingler, Laura Calo, Rebecca Treacy, Tamas Revesz, Malin Parmar, Michel Goedert, Miratul M. K. Muqit, Maria Grazia Spillantini

**Affiliations:** 1 Cambridge Centre for Brain Repair, University of Cambridge, Cambridge, UK; 2 Department of Experimental Medical Science and Lund Stem Cell Center, Lund University, Lund, Sweden; 3 Queen Square Brain Bank for Neurological Disorders, Department of Molecular Neuroscience, Institute of Neurology, University College London, London, UK; 4 Department of Neurology, Ninewells Hospital, College of Medicine, Nursing and Dentistry, University of Dundee, Dundee, UK; 5 Genetics Laboratories, Molecular Genetics, Addenbrooke’s Treatment Centre, Addenbrooke’s Hospital, Cambridge, UK; 6 Medical Research Council, Laboratory of Molecular Biology, Cambridge, UK; 7 Medical Research Council Protein Phosphorylation and Ubiquitylation Unit, College of Life Sciences, University of Dundee, Dundee, UK

**Keywords:** K298E MAPT mutation, Tauopathies, Human induced-neurons

## Abstract

Frontotemporal lobar degeneration (FTLD) consists of a group of neurodegenerative diseases characterized by behavioural and executive impairment, language disorders and motor dysfunction. About 20–30 % of cases are inherited in a dominant manner. Mutations in the microtubule-associated protein tau gene (*MAPT*) cause frontotemporal dementia and parkinsonism linked to chromosome 17 (FTDP-17T). Here we report a novel *MAPT* mutation (K298E) in exon 10 in a patient with FTDP-17T. Neuropathological studies of post-mortem brain showed widespread neuronal loss and gliosis and abundant deposition of hyperphosphorylated tau in neurons and glia. Molecular studies demonstrated that the K298E mutation affects both protein function and alternative mRNA splicing. Fibroblasts from a skin biopsy of the proband taken at post-mortem were directly induced into neurons (iNs) and expressed both 3-repeat and 4-repeat tau isoforms. As well as contributing new knowledge on MAPT mutations in FTDP-17T, this is the first example of the successful generation of iNs from skin cells retrieved post-mortem.

## Introduction

Frontotemporal lobar degeneration (FTLD) consists of a group of disorders that manifest as the result of an initial pathological damage of the frontal and temporal lobes of the cerebrum [2, 19, 21]. Hereditary forms represent 20–30 % of all cases of FTLD and up to 15–20 % of these carry mutations in *MAPT,* the gene coding for the microtubule-associated protein tau [22]. Patients carrying *MAPT* mutations are characterized by filamentous and hyperphosphorylated tau inclusions in neuronal and glial cells, causing neurodegeneration. In normal brain, tau promotes microtubule assembly and stabilization, and is involved in axonal transport [10]. In adult human brain six tau isoforms are produced by alternative mRNA splicing from a single gene located on chromosome 17 [7]. They differ by the presence of 29- or 58- amino acid inserts in the amino-terminal half of the protein, and by the presence of three (3R) or four (4R) repeats in the carboxyl-terminal half. The repeats and some adjoining sequences constitute the microtubule-binding domains of tau [12]. *MAPT* mutations have their primary effect either at the protein level, reducing the ability of tau to interact with microtubules, or at the mRNA level, altering the balance between 3R and 4R tau isoforms [6, 10]. Here we report the identification and characterization of a novel *MAPT* mutation in exon 10, 892 AAA›GAA, leading to a change from lysine to glutamate at codon 298 (K298E). This mutation significantly reduced the ability of tau to promote microtubule assembly and altered exon 10 splicing by increasing the expression of 4R tau. In the proband’s brain, neuronal and glial 4R tau pathology was present. Moreover induced neurons (iNs) obtained via transcription-factor mediated direct conversion from patient skin-derived fibroblasts with the K298E mutation expressed both 3R and 4R tau isoforms. This expression pattern was similar to that of neurons from an age-matched control, but differed from that of neurons obtained from embryo-derived human fibroblasts.

## Materials and methods

### Case report

The proband was referred at the age of 67 because of progressive gait difficulties and non-fluent aphasia. Her symptoms had rapidly progressed over the preceding 2 years. Her father had been diagnosed with Alzheimer’s disease in his 60 s and had died at the age of 67. The only living relative is the proband’s son who is currently asymptomatic. The proband died at the age of 68 and her brain was donated to the Queen Square Brain Bank for Neurological Disorders, UCL Institute of Neurology.

### Genetic analyses

Genomic DNA was extracted from 5 ml of blood collected from the patient and it was analysed at the East Anglian Regional Molecular Genetics Laboratory, Addenbrooke’s Hospital, Cambridge UK. Fluorescence sequencing of exons 1–5, 7, 9–13 (including intronic boundaries) of MAPT gene was performed.

### Purification of recombinant tau

The quick change lightning site-directed mutagenesis kit (Stratagene, Edinburgh, UK) was used to introduce the K298E mutation into the shortest 4R isoform (4R0N, T43) of human brain tau.

Wild-type, P301S and K298E mutant human tau cDNAs were subcloned into pRK172 and transformed into BL21 (DE3) *E. coli*, as described [8]. Briefly, following induction with 0.4 mM IPTG, the cells were collected by centrifugation, the pellet was resuspended in 30 ml of 25 mM Tris pH 7.4, 10 mM EDTA, 0.1 mM DTT, 0.1 mM PMSF and the cell suspension disrupted at 4 °C in a constant system cell disruptor. Recombinant tau protein was purified by anion-exchange chromatography and dialysed overnight at 4 °C against 40 mM HEPES, pH 7.4, plus 0.1 mM DTT.

### Microtubule assembly assay

The ability of recombinant tau proteins to promote microtubule assembly was monitored by turbidity, as described [8]. Tubulin (Cytoskeleton Inc., Denver, CO, USA) was dissolved in G-PEM buffer (80 mM Na-PIPES pH 6.9, 1 mM MgCl_2_, 1 mM EGTA, 1 mM GTP) and mixed with 77.5 μl of G-PEM. Wild-type or mutant tau was then added and the mixture was quickly transferred to a quartz cuvette. The final concentrations of both tubulin and tau were 1 mg/ml. Polymerization of microtubules was monitored by measuring the absorbance at 350 nm at 37 °C over 10 min. All experiments were performed in triplicate.

### Minigene transfection

A previously described [1] *MAPT* minigene construct encompassing exons 9–11 and flanking E10 intronic sequences in pcDNA3 was kindly provided by Dr R. Vuono, as was the construct with the I10+3 G>A intronic mutation. The quick change lightning site-directed mutagenesis kit was used to introduce the K298E mutation in exon 10 of *MAPT*. Neural stem cells (NSCs) were obtained from human embryonic stem cells and maintained in a neural precursor stage in defined medium in the presence of 20 ng/ml of FGF2, as described [15, 24]. Transient transfections were performed using Lipofectamine 2000, according to the manufacturer’s instructions (Invitrogen, Eugene OR, USA). Cells were plated into a six-well plate at a density of 2 × 10^5^ cells/well and incubated in the absence of antibiotics for 24 h. For single transfections, 2 μg of the wild-type or mutant minigene DNA was mixed with 10 μl Lipofectamine 2000 in Optimem medium (Invitrogen, Eugene OR, USA) and incubated for 20 min at room temperature. For co-transfections, 1 μg of each construct (wild-type and mutant) was used. Cells were exposed to the transfection mixture for 5 h at 37 °C and 5 % CO_2_ and returned to their growing medium. To analyse minigene expression, cells were harvested 48 h after transfection.

### RNA extraction and RT-PCR

Total RNA was extracted from NSCs with the RNeasy mini kit (Qiagen, Crawley, UK). RNA concentration and purity were estimated using spectrophotometry (absorbance at 230, 260 and 280 nm). Three-repeat and 4R tau mRNA expression was detected by RT-PCR using the onestep RT-PCR kit (Qiagen, Crawley, UK) and the following primers: exon 9 forward 5′ CTCCAAAATCAGGGGATCGC 3′ and exon11 reverse 5′ TATGATGGATGTTGCCTAATGAGCC 3′. Bands corresponding to mRNAs with and without exon 10 were separated by electrophoresis on 1 % agarose gels. The same method was used for RT-PCR of RNA extracted from frontal cortex of the patient with the K298E mutation and of a control subject.

### Human brain tissues

Tissue from cerebral cortex of a patient with the I10+3 G>A MAPT mutation was obtained from the Brain Bank of the Indiana University Alzheimer Centre [26]. Frontal cortex from an age-matched control subject was obtained from the Cambridge Hospital Brain Bank. The work on human tissue was covered by Cambridge LREC ethical approval (ref no 09/40). Neuropathological investigation was performed at the Queen Square Brain Bank for Neurological Disorders, London. Handling of human tissue was according to the UK Human Tissue Act 2006. Approval for “Generation of patient-specific stem cells for research in neurodegenerative disorders of the CNS” was granted by the Hertfordshire Research Ethics Committee (ref no 09/H0311/88).

### Immunohistochemistry

Following fixation in 10 % buffered formalin, the left half of the proband’s brain was sliced in the coronal plane and blocks were selected for histological examination. The right half of the brain was sliced in the coronal plane, flash frozen and stored at −80 °C. Paraffin-embedded sections (8 μm) from cortical, subcortical, brainstem and cerebellar regions were stained using haematoxylin eosin (HE) and Gallyas silver impregnation. Immunohistochemistry was performed using the antibodies listed in Table 1. Sections were deparaffinised using xylene and rehydrated using graded alcohols. Endogenous peroxidase activity was blocked with methanol/0.3 % H_2_O_2_. Antigen retrieval was achieved by heating sections in a pressure cooker for 10 min in citrate buffer (pH 6.0). For Aβ and α-synuclein immunohistochemistry, sections required pre-treatment with formic acid for 10 min prior to antigen retrieval. Sections were incubated in dried milk solution (10 % in PBS, 30 min) to block non-specific binding. Primary antibodies diluted in PBS were applied for 60 min at room temperature, followed by either biotinylated anti-rabbit IgG or biotinylated anti-mouse IgG (1:200, 30 min; DAKO) and ABC complex (30 min; DAKO). The sections were developed with di-aminobenzidine/H_2_O_2_ and counterstained with HE.

**Table 1:** Antibodies used in this study

Antibody	Epitope/antigen	Species	Dilution	Source
AT8	Tau (phospho-Ser202/Ser205)	Mouse	1:600	Autogen Bioclear, Wiltshire, UK
RD03	3-Repeat tau	Mouse	1:2,000	Dr Rohan De Silva/Millipore
RD04	4-Repeat tau	Mouse	1:100	Dr Rohan De Silva/Millipore
AT100	Tau (phospho Ser212/Thr214/T217)	Mouse	1:200	Autogen Bioclear, Wiltshire, UK
Aβ	β-amyloid protein (M0872)	Mouse	1:100	DAKO, Ely, UK
α-Synuclein	α-Synuclein	Mouse	1:75	Novocastra, Peterborough, UK
p62	p62 lck ligand	Mouse	1:100	BD Transduction Labs, Oxford, UK
TDP-43	Tar DNA-binding protein-43	Mouse	1:800	Abnova, Taipei City, Taiwan
GFAP	Glial fibrillary acidic protein	Rabbit	1:1 K	DAKO, Ely, Uk
Neurofilaments	Phosphorylated (SMI 31) and non-phosphorylated (SMI 32) neurofilaments	Mouse	SMI 31—1:5,000 SMI 32—1:500	Sternberger Monoclonals Inc, Baltimore, USA
αB crystallin	Novacastra	Mouse	1:300	Novocastra, Peterborough, UK

Neuronal loss and gliosis were assessed on HE-stained sections and scored semi-quantitatively as follows: − = none; + = mild; ++ = moderate; +++ = severe. The frequency of tau-positive inclusions of different types was assessed using antibody AT8 and scored semi-quantitatively: − = none; + = infrequent; ++ = moderate; +++ = frequent.

### Immunocytochemistry

Cells were fixed with 4 % paraformaldehyde and pre-incubated for 1 h in blocking solution (5 % normal goat serum, 0.02 % Tween in PBS). Primary antibodies (monoclonal 3R tau isoform 1:500, Millipore; monoclonal 4R tau isoform, 1:500 Millipore; polyclonal betaIIItubulin 1:1,000, Covance) were incubated overnight at 4 °C. For 3R and 4R tau immunofluorescence, cells were incubated with biotinylated secondary antibodies (1:250) for 2 h at room temperature and 1 h with fluorescent streptavidin (Alexa Invitrogen, 1:500). Anti rabbit fluorescent-conjugated secondary antibodies (Alexa, Invitrogen Molecular Probes) diluted 1:1,000 were used for betaIIItubulin staining. Double-labelling immunofluorescence was performed by sequential incubation with the appropriate primary antibodies. Cell nuclei were visualized with Hoechstbye (Sigma Aldrich, 1:5,000) as previously reported [15]. Fluorescence was visualized using a Leica TMDM 6000B fluorescence microscope.

### Immunoblotting

Soluble tau was extracted with perchloric acid as described [8] from the frontal cortex of the proband, from the frontal cortex of a patient with the I10+3 G>A intronic *MAPT* mutation [26] and from the frontal cortex of a control subject. Insoluble tau was extracted with sarkosyl from the frontal cortex of the proband, as described [9]. Soluble and sarkosyl-insoluble tau proteins were dephosphorylated with 0.3 U/μl alkaline phosphatase from *E. coli* (Sigma-Aldrich) in 50 mM Tris–HCl and 5 mM MgCl_2_ for 4 h at 65 °C. The proteins were separated on 10 % SDS-PAGE and transferred onto immunobilon-P (Millipore, Bedford, MA, USA). The membranes were incubated for 1 h with 5 % milk w/v (Premier International Foods, Spalding, UK) in TBS/0.01 % Tween-20. They were then probed with a polyclonal anti-human tau serum (Dako, Glostrup, Denmark, 1:1,000), followed by peroxidase-conjugated secondary antibody (Dako, 1:10,000). The blots were developed using Western lightning chemiluminescence reagent Plus (Perkin Elmer, Waltham, MA, USA).

### Derivation and transdifferentiation of human fibroblasts

A skin biopsy was taken from the proband 2 h after death. It was cultured as an explant in DMEM supplemented with 10 % FCS and 1 % penicillin/streptomycin for 2 weeks until fibroblasts started to appear. The fibroblasts were then expanded and transduced during their second passage as described previously [25] with the addition of delaying doxycycline administration (=transgene activation) by 5 days. As controls we used human embryonic fibroblasts (hEFs) and human adult fibroblasts (hAFs) transduced in the same way.

## Results

### Patient clinical data

In 2009, a 67-year-old lady was referred to the neurology service for investigation of progressive gait difficulties and non-fluent aphasia. She had previously developed a tremor of her right hand at the age of 48, which had been non-troubling. At the age of 65, the tremor became more severe and also spread to involve the left hand such that she had to give up writing and also driving. At that time her MMSE score was 27/30 and a CT brain scan revealed bilateral cortical atrophy. SPECT imaging by [(123) I] FP-CIT (DaTscan) was normal. Over the next 2 years, her condition progressed and she suffered a decline in her balance and mobility with initially recurrent falls and then progressing such she could no longer stand or sit unaided. She also exhibited a decline in her speech with severe word finding difficulties. This was associated with marked anxiety and cognitive decline. She had now become dependent for all her daily living activities. During this time there had been no fluctuation in her condition and no history of visual hallucinations. Her past medical history included sick sinus syndrome and she had been fitted with a cardiac pacemaker. The family history was relevant as her father was diagnosed with Alzheimer’s disease in his 60 s and died at the age of 67. There were no other family members with dementia. She was a non-smoker and there was no history of alcohol excess.

Significant examination findings included: an unreactive facies and severe cognitive impairment, which prevented completion of a full MMSE. There was a paucity of speech and her speech was effortful. She was able to register three objects but could not recall any. She was poor at naming objects. She could perform a one-stage command but not three. She exhibited perseveration. She had prominent primitive reflexes including grasp reflexes and rooting response. In her limbs, she had a bilateral symmetrical myoclonic tremor (arms > legs), which was irregular and jerky. She had increased tone in all limbs with marked spasticity of her right arm. Her deep tendon reflexes were brisk (right > left) with down-going plantar responses.

Blood investigations, including treponemal serology and vasculitic screen, were unremarkable. A repeat CT brain scan showed progression of the cerebral atrophy. CSF analysis was normal. EEG revealed intermittent slow waves, mainly in the right hemisphere, suggesting cortical dysfunction. Genetic analysis of MAPT revealed a novel single nucleotide change from A>G (AAA to AAG) at codon 298 in exon 10 which resulted in a change from Lys 298 to Glu in one allele. The proband’s condition progressed and she died in 2010.

### Microtubule assembly

**Fig. 1:**
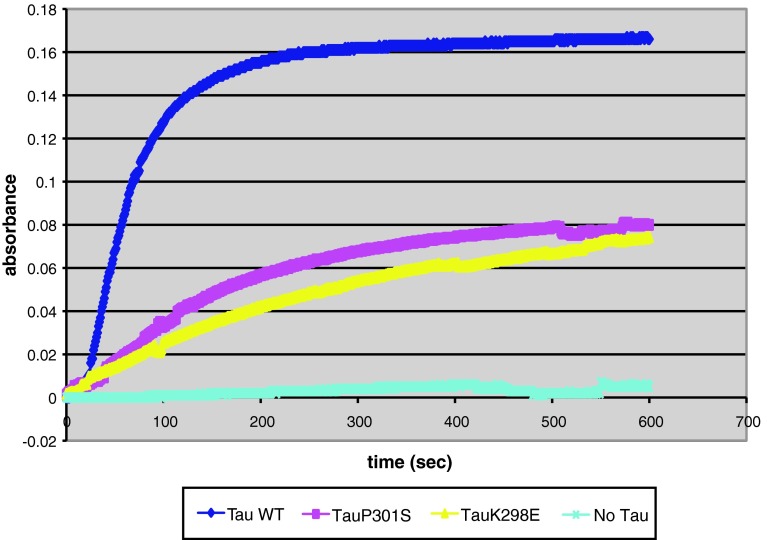
Microtubule assembly assay. Polymerization of tubulin induced by wild-type tau, P301S tau and K298E tau. Microtubule assembly was monitored over time by optical density (absorbance 350 nm). The results are expressed as the means of three different experiments

Microtubule assembly assays were performed with recombinant wild-type or mutant tau proteins carrying the K298E mutation or the previously characterized P301S mutation. Both P301S and K298E recombinant proteins showed a reduced ability to promote microtubule assembly compared to wild-type tau (Fig. 1), suggesting that the K298E mutation disrupts the ability of tau to interact with microtubules.

### Tau splicing

**Fig. 2:**
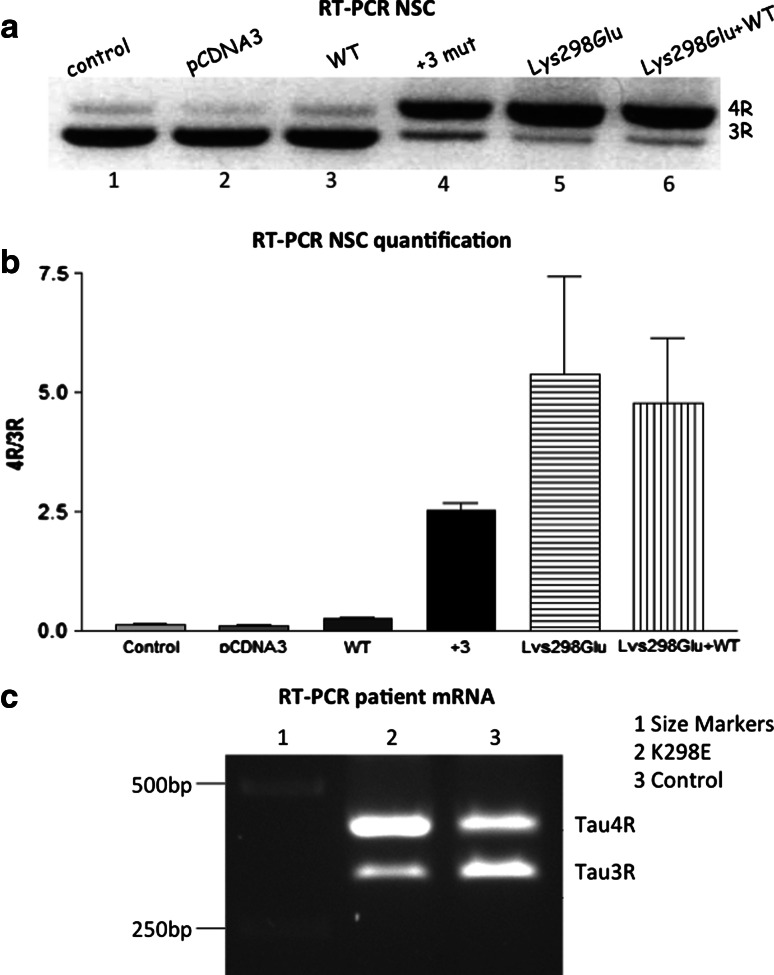
Reverse transcription polymerase chain reaction (RT-PCR) analysis of tau mRNA expression in human neural stem cells transfected with a tau minigene and mRNA extracted from frontal cortex of the K298E patient. The tau minigene construct consisted of the pcDNA3 vector containing tau exons 9, 10 and 11, and the E10 flanking intronic sequences of minimal length for correct splicing. The splicing patterns of *MAPT* exon 10 in minigene transfected human neural stem cells (NSCs) are shown. **a** Untransfected human NSCs expressed high levels of 3R tau mRNA and low levels of 4R tau mRNA (*lane 1*) similar to cells transfected with the empty pcDNA3 vector (*lane 2*). Transfection with the wild-type tau minigene did not change the ratio between the two mRNAs (*lane 3*), with the expression level of 3R tau mRNA remaining predominant. When human NSCs were transfected with the minigene carrying the +3 *MAPT* mutation, the mRNA level of 4R tau increased (*lane 4*) and this also occurred after transfection with the minigene carrying the K298E mutation (*lane 5*). Co-transfection of wild-type and K298E minigenes did not alter the ratio between 3R and 4R tau isoforms mRNAs (compared to K298E alone), indicating that the K298E mutation has a dominant effect on exon 10 splicing (*lane 6*). The quantification shown in **b** was the result of three different transfection experiments and was performed using the Image J program (http://imagej.nih.gov/). **c** RT-PCR of mRNA extracted from frontal cortex of the K298E mutation patient showed a clear increase in 4R tau mRNA expression and a decrease in 3R tau mRNA expression compared to mRNA extracted from an age-matched control subject

We next examined the effect of the K298E tau mutation on exon 10 alternative mRNA splicing in cultured cells. A minigene construct with A892>G mutation was engineered based on the previously described human tau minigene model [17]. Human NSCs were transiently transfected at day 20 of differentiation with mutant K298E or the wild-type tau minigene, as described [1]. Untransfected human NSCs expressed a strong 3R tau isoform and a very weak 4R tau band at the mRNA but not the protein levels as previously shown [15] (Fig. 2a, lane 1). When transfected with the empty vector or the wild-type tau minigene, the level of expression of 3R tau remained high. In contrast, transfection of the I10+3 mutation minigene (Fig. 2a, lane 4) or the K298E mutant minigene (Fig. 2a, lane 5) altered the ratio between 3R and 4R tau isoforms in favour of 4R tau, indicating that the two mutations strongly reduced the splicing out of exon 10. Co-transfection of the wild-type minigene and the K298E minigene still increased the amount of transcripts with exon 10, indicating that the K298E mutation had a dominant effect on the splicing of exon 10 (Fig. 2a, lane 6). Figure 2b shows a quantification of tau isoform mRNA levels in human NSCs in three different experiments for each line. The effect of the K298E on exon 10 splicing was confirmed by RT-PCR of RNA extracted from the patient’s brain. A clear increase of the 4R tau band was observed compared to the RT-PCR of RNA extracted from the cortex of a control subject (Fig. 2c).

### Brain post-mortem study

The left half of the brain weighed 513 g. External examination and coronal slices showed severe atrophy of the frontal lobe, including the motor strip, and the anterior temporal lobe, while the parietal and posterior temporal lobes were less severely affected and there was good preservation of the occipital lobe. There was severe dilatation of the frontal and temporal horns of the lateral ventricle. The cortical ribbon was thin throughout the frontal lobe and in all three temporal gyri anteriorly with patchy thinning in the parietal lobe and in the second and third temporal gyri posteriorly. There was atrophy of the caudate nucleus and thalamus with preservation of the putamen, globus pallidus and subthalamic nucleus. The hippocampus and amygdala were severely atrophic. Both the substantia nigra and the locus coeruleus were pale. Medulla and cerebellum were unremarkable.

### Histology and immunohistochemistry

Immunohistochemistry showed widespread neuronal loss and gliosis with abundant deposition of hyperphosphorylated tau in neurons and glia. The semiquantitative assessment of these features in different brain regions is detailed in Table 2.

**Table 2:** Semi-quantitative assessment and regional distribution of neuronal loss, gliosis and tau pathology

	Neuronal loss	Gliosis	Tau pathology							
			Threads	Dots	Neuronal rings/crescents	Neuronal flame shaped	Neuronal globular	Neuronal diffuse	Oligodendroglial	Astrocytic
Cortex										
Frontal	++	+++	+++	+	+++	+	++	++	+	++
Motor	+	++	+++	+	+++	++	+	+++	+	+
Temporal	+	+	+++	+	+++	+	+	++	+	++
Parietal	++	+++	+++	+	+++	+	++	++	+	++
Occipital	−	+	++	+	+	++	+	+	−	+
Cingulate	+	++	+++	+	+++	+	+	+++		+
Sub-cortical white matter										
Frontal	N/A	+++	++	++	N/A	N/A	N/A	N/A	+++	−
Motor	N/A	+++	++	+	N/A	N/A	N/A	N/A	+++	−
Temporal	N/A	+	++	++	N/A	N/A	N/A	N/A	+++	−
Parietal	N/A	+++	++	++	N/A	N/A	N/A	N/A	+++	−
Occipital	N/A	++	+	+	N/A	N/A	N/A	N/A	+	−
Cingulate	N/A	++	++	++	N/A	N/A	N/A	N/A	+++	−
Amygdala	+	+++	++	+++	+++	+	++	+++	+	++
Hippocampus										
Dentate fascia	− (Dispersed)	+	++	+	++	+	−	+++	−	−
CA4	+++	+++	+++	+	+	−	−	+	+	−
CA3	+++	+++	+++	+	−	−	−	+	+	−
CA2	−	++	+++	+	−	−	+	+++	−	−
CA1	+	++	+++	+	+	++	+	+++	+	−
Subiculum	+	++	+++	+	+	+	++	+++	+	+
Entorhinal cortex	+	+++	+++	++	+++	+	+	+++	+	+
Transentorhinal cortex	+	+++	+++	++	+++	−	++	+++	+	+
Caudate	++	++	+++	+	+++	−	++	++	+	++
Putamen	−	++	+++	+	+++	−	++	+	+	++
Globus pallidus	−	+++	++	+	+	−	+	+	+++	−
Meynert	−	++	+++	++	−	−	+++	+++	+	+
Thalamus	−	++	+++	+	−	−	+	++	+++	+
Subthalamic nucleus	−	++	++	+	−	−	++	++	+	−
Midbrain tegmentum	−	++	++	+	+	−	+	+++	+	−
Substantia nigra	+	++	+++	+	+	−	+	+++	+	−
Locus coeruleus	+	++	+	++	−	−	−	++	−	−
Pontine tegmentum	−	++	++	++	−	−	+	+++	+	−
Pontine nuclei	−	++	+	+++	−	−	+	++	++	−
Dorsal motor nucleus of vagus	−	++	+	+	−	−	+	+	−	−
Twelfth nerve nucleus	−	++	+	++	−	−	−	−	+	−
Inferior olive	−	++	−	+++	−	−	−	−	−	+
Cerebellar Purkinje cells	+	+	−	+	−	−	−	−	−	−
Cerebellar white matter	N/A	++	−	+	N/A	N/A	N/A	N/A	−	−
Dentate nucleus	−	+	+	+	−	−	+	+	−	−

*N/A* not applicable

**Fig. 3:**
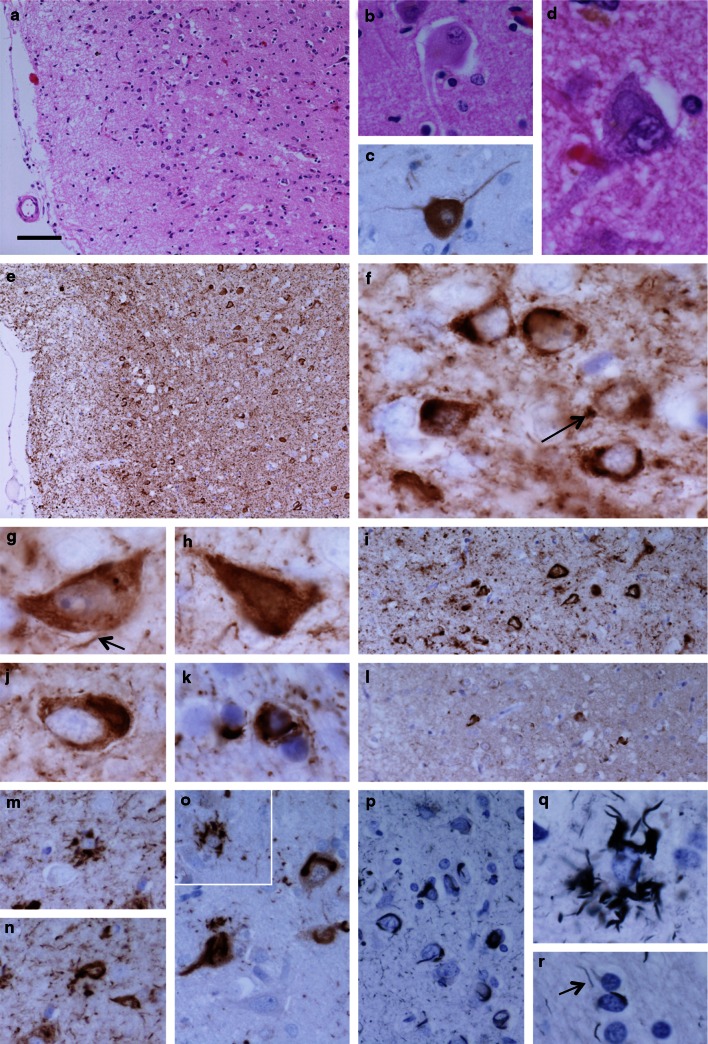
Neuropathological features in the anterior frontal cortex of the proband. There was mild superficial spongiosis (**a**), ballooned neurons were identified in H&E stained sections (**b**) and were confirmed by αB crystallin immunohistochemistry (**c**). A proportion of neurons contained basophilic inclusions (**d**). Tau-immunoreactive inclusions were abundant (**e**). High magnification revealed tau-positive dots and threads (**f** *arrow* and **g** *arrow*, respectively) and frequent neuronal inclusions with ring or crescent morphology (**f**), diffuse (**g**), flame-shaped (**h**) or globular appearance (**j**). In the subcortical white matter tau-immunoreactive oligodendroglial inclusions and threads were abundant (**k**). All inclusion types showed strong immunoreactivity for 4R tau isoforms (**i**), including structures resembling tufted astrocytes (**m**) and neuronal inclusions (**n**), while only a minority of inclusions could be identified using immunohistochemistry for 3R tau (**l**). There was strong immunoreactivity for p62 in a proportion of neuronal (**o**) and astrocytic inclusions (**o** *inset*). Many neuronal (**p**), astrocytic (**q**) and oligodendroglial inclusions (**r**) in addition to threads (**r** *arrow*) were argyrophilic as demonstrated using the Gallyas silver impregnation method. **a**, **b**, **d** H&E; **c** αB crystallin immunohistochemistry; **e**–**k** phospho-tau immunohistochemistry (AT8); **i**, **m**, **n** 4R tau immunohistochemistry; **l** 3R tau immunohistochemistry; **o** p62 immunohistochemistry; **p**–**r** Gallyas silver impregnation. *Bar* in **a** represents 100 μm in **a**, **e**; 50 μm in **i**, **l**; 25 μm in **b**, **c**, **m**–**p**; 10 μm in **d**, **f**–**k**, **q**, **r**

**Fig. 4:**
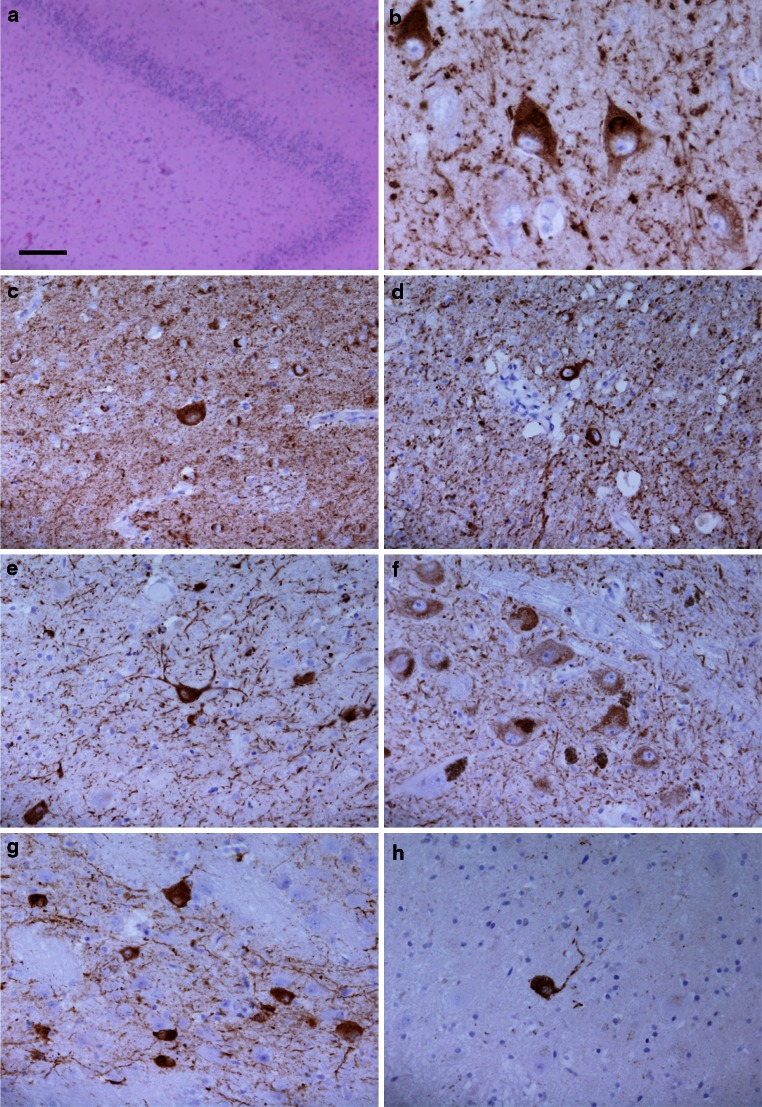
Neuropathological features in the hippocampus and subcortical nuclei of the proband. In the hippocampal formation there was marked neuronal loss in the CA4 subregion and dispersion of the granule cells of the dentate fascia (**a**) and tau immunohistochemistry showed widespread neuronal immunoreactivity, often diffuse in character, and sometimes with additional filamentous inclusions (**b**, subiculum). Tau-immunoreactive inclusions were widespread in subcortical structures, including: caudate nucleus (**c**), globus pallidus (**d**), subthalamic nucleus (**e**), substantia nigra (**f**), pontine nuclei (**g**) and dentate nucleus (**h**). **a** H&E; **b** 4R tau immunohistochemistry; **c**–**h** phospho-tau immunohistochemistry (AT8). *Bar* in **a** represents 250 μm in **a**; 50 μm in **c**–**h**; 25 μm in **b**

In addition to cortical thinning, gliosis and mild superficial spongiosis in the frontal, temporal and parietal cortices, a number of ballooned neurons were observed, they were particularly prominent in the cingulate gyrus (Fig. 3a–c). Neuronal loss was most severe in the frontal and parietal cortices, the caudate nucleus and in the CA3 and CA4 subregions of the hippocampus (Fig. 4a). Although neuronal loss was not marked in the dentate fascia of the hippocampus, there was dispersion of the granule cell layer (Fig. 4a). Poorly defined basophilic inclusions were evident in a proportion of neurons in haematoxylin- and eosin-stained sections (Fig. 3d). Tau immunohistochemistry revealed extensive neuronal and glial deposition of phosphorylated tau (AT8, epitope phosphoSer202 and phosphoThr205) (Figs. 3e–k, 4c–h). Neuronal inclusions of various types were observed: crescentic or ring-like perinuclear inclusions; diffuse cytoplasmic staining, sometimes also containing fine filaments; flame-shaped inclusions and globular inclusions. The former were particularly marked in the small neurons of the superficial cortical laminae (Fig. 3f–j). Astrocytic tau pathology was observed with morphological features similar to tufted astrocytes (Fig. 3m, o, q). In white matter there were frequent tau-immunoreactive oligodendroglial inclusions, numerous threads and dots (Fig. 3k). All of these structures were immunoreactive for 4R tau and astrocytic pathology was most clearly visible in this preparation (Figs. 3l, m, n; 4b). 3R tau was also detected in a proportion of inclusions, predominantly neuronal, using immunohistochemistry, but immunoreactivity was much more restricted than that of 4R tau (Fig. 3l). AT100-positive staining recognized a proportion of neuronal inclusion and threads. P62 immunohistochemistry highlighted abnormal astrocytes, some of the neuronal inclusions and a proportion of oligodendroglial inclusions in white matter (Fig. 3o). Ubiquitin immunohistochemistry revealed weak immunoreactive inclusions mostly with a morphology compatible with astrocytic inclusions. Gallyas silver staining demonstrated a proportion of neuronal, astrocytic and oligodendroglial inclusions, in addition to some of the thread pathology indicating fibrillar tau deposition (Fig. 3p–r). There was no TDP-43 or Aβ pathology in neocortical regions or the hippocampus. No α-synuclein pathology in the form of Lewy bodies, Lewy neurites or glial cytoplasmic inclusions was found in brainstem, limbic or cortical regions.

### Immunoblotting

**Fig. 5:**
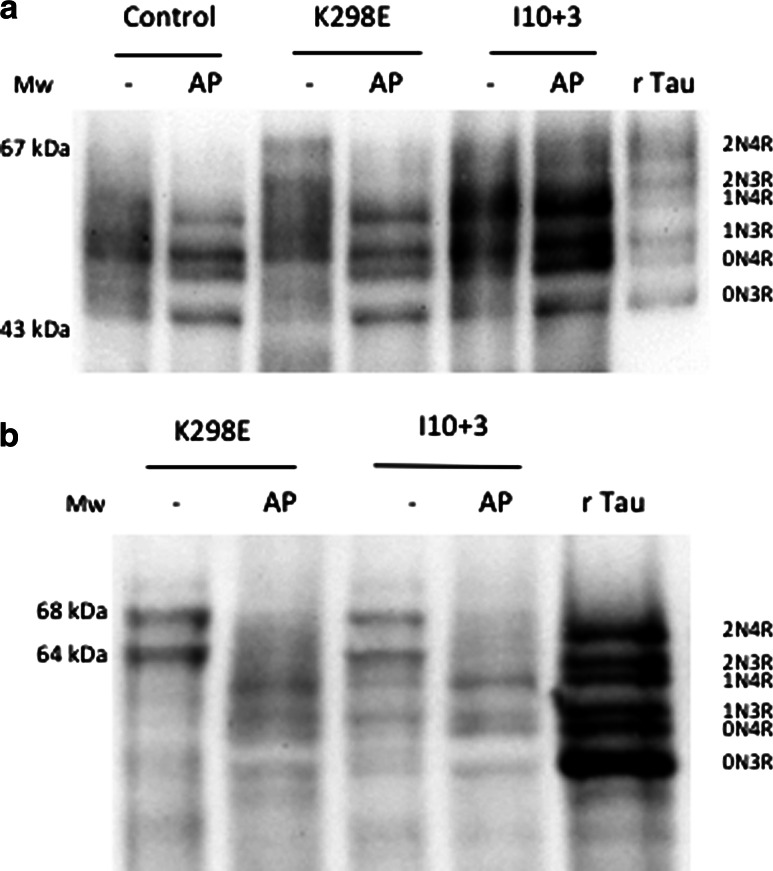
Soluble and insoluble tau protein from human brain. **a** Tau isoforms extracted from frontal cortex of brains of the patient with the K298E MAPT mutation, a patient with the I10+3 G>A (+3) MAPT mutation and a control subject before (−) and after (AP) dephosphorylation with alkaline phosphatase (AP). The pattern of tau isoforms extracted from the frontal cortex of the patient with the K298E mutation shows that this mutation induces an increase in 1N4R tau isoform expression, with a decrease in 1N3R tau isoform. A slight increase can be also observed for the 0N4R tau isoform. As comparison extracts from cortex of a patient with the I10+3 G>A (+3) MAPT mutation and a control subject before and after AP treatment are also shown. Recombinant human tau (rTau) isoforms were run as a control. Molecular weight markers (*M*_w_). **b** Sarkosyl-insoluble tau from the frontal cortex of the patient with the K298E tau mutation before (−) and after (AP) dephosphorylation with alkaline phosphatase and from frontal cortex of a patient with the I10+3 G>A (+3) mutation. The immunoblot shows that insoluble tau from frontal cortex contained mainly 4R tau isoforms although some 3R tau appears also to be present in the K298E case. The extra bands in the +3 extract could be degradation products in a tissue that also contained some Alzheimer type tau changes. Recombinant tau isoforms (rTau) were run in parallel to the samples. Molecular weight markers (*M*_w_)

To measure the level of expression of tau isoforms, soluble tau was extracted with perchloric acid from the frontal cortex of a patient with the I10+3 mutation, normal adult human brain and the frontal cortex of the proband with the K298E mutation. All six tau isoforms were expressed in the normal, I10+3 and K298E tau mutant brains (Fig. 5a); however, in the K298E case the 4R tau isoforms 1N4R appeared increased and the 3R tau isoforms 1N3R decreased (Fig. 5a). A slight increase in the 0N4R tau isoform could also be observed in the K298E case. These data showed and confirmed that tau pathology in the frontal cortex had a high prevalence of 4R tau isoforms. Sarkosyl-insoluble tau extraction from the frontal cortex of the proband with the K298E *MAPT* mutation confirmed that 4R tau was the major component, although some 3R tau was also present (Fig. 5b). Sarkosyl-insoluble tau extracted from the brain of the I10+3 case, where filaments had been shown to contain 4R tau, is shown for comparison.

### Tau isoforms expression in K298E-induced neurons

**Fig. 6:**
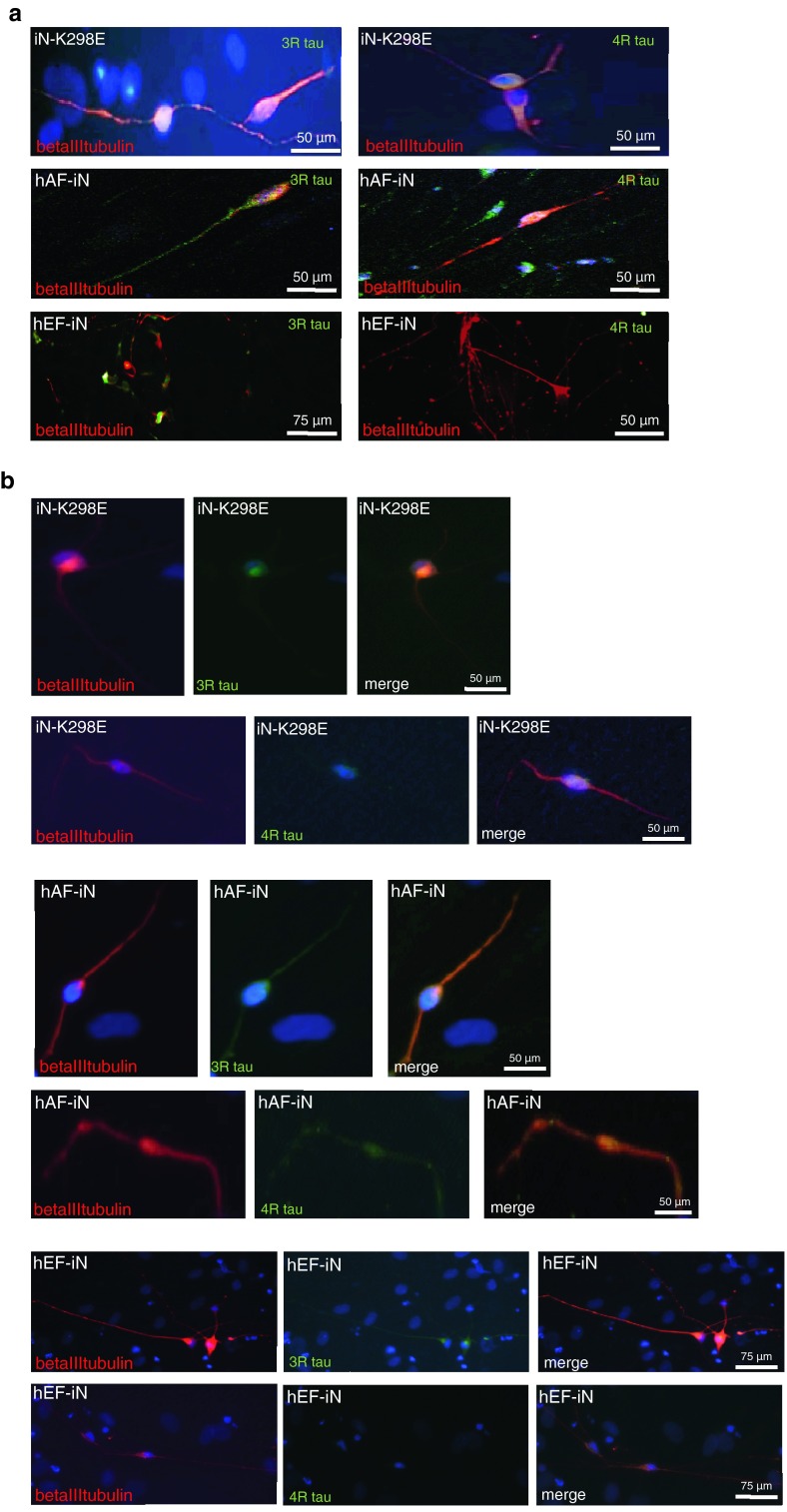
Tau isoform expression in human K298E-derived neurons. **a** 3R and 4R tau isoform expression in induced neurons with K298E mutation (iN-K298E), in induced neurons from human adult fibroblasts (hAF-iN) and in induced neurons from human embryonic fibroblasts (hEF-iN) at 30 days after transgene activation. **b** 3R and 4R tau isoform expression in iN-K298E, hAF-iN and hEF-iN at 53 days post-transgene activation and beginning of differentiation

Direct conversion of human fibroblasts into neurons is of potential clinical utility and of interest for neurological disease modelling, as well as for cell therapeutics [3]. A skin biopsy was taken from the patient with the K298E mutation 2 h post-mortem and fibroblasts were derived. These were then expanded and converted into iNs, as previously described [24, 25]. As controls, human embryonic fibroblasts (hEFs) and human adult fibroblasts (hAFs) were converted into neurons following an identical protocol. Two weeks after transgene activation, induced neurons started to appear in both controls and K298E cells, but tau was not detected (data not shown). Thirty days after transgene activation, while iNs obtained from human embryonic fibroblasts (hEF-iN) expressed only the 3R tau isoform, iNs with the K298E mutation (iN-K298E) and iNs from hAF (hA-iN) expressed both 3R and 4R tau (Fig. 6a). Fifty-three days after transgene activation, the level of expression of both tau isoforms in the iN-K298E did not increase (Fig. 6b) and was comparable to the level of tau observed in hAF-iNs. However, hEF-iNs still expressed only 3R tau 53 days after transduction (Fig. 6b). The limited availability of fibroblasts and differentiated neurons did not allow for biochemical studies or more indepth analyses.

## Discussion

We report a novel missense mutation in *MAPT* in a 67-year-old lady who was referred with dementia and non-fluent aphasia. Genetic analysis revealed the presence of a K298E missense mutation in exon 10 in one of her alleles.

Missense mutations in *MAPT* have mainly been identified in the coding region, with a cluster of mutations close to the 5′ splice site of exon 10. Exon 10 mutations and intronic mutations immediately downstream of the 5′ splice site of exon 10 can modulate the splicing of tau pre-mRNA, which leads to an over-representation of 4R tau. However, some exon 10 mutations, including P301L and P301S, only have effects at the protein level [13, 14, 20]. The region of exon 10, which contains Lys 298 has been shown to contain an exonic splice suppressor (ESS) that negatively regulates the splicing of exon 10 [4, 5]. Indeed, mutations at codon 296, in the same ESS, have been described, including N296H, N296N, and ΔN296 [16, 23, 27]. Interestingly, N296N and N296H lead to an increase in 4R tau mRNA, while mutation ΔN296 has no effect on alternative mRNA splicing of *MAPT* [11, 28]. In contrast, the ΔN296 mutation effected a marked reduction in microtubule assembly while the N296H mutation also significantly reduced microtubule assembly, but less so ΔN296 [12]. The K298E mutation similar to the N296H, affects both tau microtubule assembly and 4R tau expression.

We performed a microtubule assembly assay using mutant K298E recombinant tau protein. The K298E mutation reduced the ability of tau to promote microtubule assembly compared to wild-type tau. We next evaluated the effects of the K298E mutation at the mRNA level and performed minigene transfection experiments. In human NSCs, the transfection of the tau minigene expressing the K298E mutation induced the overexpression of 4R tau (Fig. 2a). Our in vitro data suggest that the K298E mutation induces the overproduction of mutant 4R tau, which is impaired in its ability to interact with microtubules. The pathogenicity of the K298E mutation was also confirmed by neuropathological analysis of the post-mortem brain, which showed severe atrophy of frontal and anterior temporal lobe, as well as of caudate nucleus and thalamus. Neuronal loss and gliosis confirmed that the pathology was widespread in frontal and parietal cortices, with abundant deposition of hyperphosphorylated tau in neurons and glia, reminiscent of progressive supranuclear palsy [18]. Tau pathology consisted mainly of 4R tau. Biochemical analysis of soluble tau showed an increase in 4R and a slight decrease in 3R tau. In sarkosyl-insoluble tau from frontal cortex of the patient, as in progressive supranuclear palsy, the 64 and 68 kDa bands contained mainly 4R tau, although a small amount of 3R tau was also present (Fig. 5).

Direct conversion of patient skin fibroblasts into neurons was performed to test whether they express tau isoforms and could show the increase in 4R tau. The skin biopsy was collected 2 h post-mortem; it was not possible to obtain induced pluripotent stem cells (iPSCs) from the biopsy-derived fibroblasts, although several attempts were made by various laboratories. Therefore, we decided to opt for direct conversion of fibroblasts into induced neurons (iNs). Although this approach gave positive results and neurons were produced, they were not in sufficient number to allow biochemical characterization of their tau isoforms. To our knowledge, this is the first study showing tau protein in iNs derived from fibroblasts of a patient with a *MAPT* mutation collected postmortem. Our analysis has given insight into the study of patient-derived iNs, i.e., that the type of controls used is critical for the evaluation of neuronal phenotype, as far as tau is concerned. We observed a difference in the time course of tau isoform expression between iNs from the proband and iNs from embryonic fibroblasts initially available and used as control, this was not present when comparing iNs from fibroblasts of the proband and of an age-matched control. This suggests that induced neurons obtained from embryonic fibroblasts can take longer to mature compared to those obtained from adult human fibroblasts and are not suitable as control.

In conclusion, we report a novel K298E tau mutation in a patient with frontotemporal dementia. The mutation affected both tau microtubule assembly and tau mRNA splicing with an increase in 4R tau. Therefore, the K298E mutation becomes another member of a small group of exon 10 mutations, which confer pathogenicity through effects at both the RNA and the protein level.
